# SHORTENING OF CLAVICLE FRACTURES: PHYSICAL *VERSUS* IMAGE EXAMINATIONS

**DOI:** 10.1590/1413-785220243202e274209

**Published:** 2024-06-24

**Authors:** Rodrigo Alves Beraldo, Caroline Izidorio Bernardes Silva, Hélio Henrique de Paiva, Ewerton Alexandre Galdeano, Renato de Moraes

**Affiliations:** 1 Instituto Jundiaiense de Ortopedia e Traumatologia (IJOT), Jundiaí, São Paulo, Brazil.; 2 Faculdade de Medicina de Jundiaí (FMJ), Jundiaí, SP, Brazil.; 3 Hospital São Vicente de Jundiaí, Nucleus of Education and Research, Jundiaí, SP, Brazil.

**Keywords:** Fractures, Bone, Clavicle, Physical examination, Radiography, Tomography, X-Ray Computed, Fraturas Ósseas, Clavícula, Exame Físico, Radiografia, Tomografia Computadorizada por Raios X

## Abstract

**Objective:**

Determine the reliability of three different methods of evaluating bone shortening in displaced midshaft clavicle fractures (DCMF).

**Method:**

A cross-sectional analytical study evaluated bone shortening by metric tape (MT), radiography (X-ray), and computed tomography (CT). Twenty-six men had been evaluated and used clavícula not broken as control. The collection of data was of the blind type for three specialists. Differences and reliability were analyzed with the Friedman and Kappa tests and validated with the T-test (CI: 95%; significance index p<0.05; Software "R" version 3.2.2).

**Results:**

The MT measurements (control) showed abnormal distribution and significant statistical difference concerning the imaging tests (p=0.000008). There was a similarity between X-ray and CT and Kappa agreement of 0.65. The fractured clavicles presented similar measurements between the three methods (p=0.059), and the T-tests proved that the similarity was caused by chance or possible measurement errors.

**Conclusion:**

Measurement by metric tape showed a tendency to overestimate bone shortening. The CT showed more reliable results for the diagnosis; however, the X-ray was sufficient for decision-making by surgeons, and therefore, it is not possible to rule out the importance of this resource for DCMF. ***Level of Evidence IV; Case-Control Study.***

## RESUMO

**Objetivo:**

Determinar a confiabilidade de três diferentes métodos de avaliação do encurtamento ósseo em fraturas deslocadas do eixo médio da clavícula (FDEMC).

**Método:**

Estudo analítico transversal que avaliou o encurtamento ósseo por fita métrica (FM), radiografia (X-Ray) e tomografia computadorizada (TC). Foram avaliados 26 homens utilizando a clavícula não fraturada como controle. A coleta de dados foi do tipo cega por três especialistas. As diferenças e a confiabilidade foram analisadas com os testes de Friedman e Kappa e validados com o teste T (IC:95%; índice de significância p<0,05; Software "R" versão 3.2.2).

**Resultados:**

As medidas de FM (controle), apresentaram distribuição anormal e diferença estatísfica significativa em relação aos exames de imagem (p=0,000008). Houve semelhança entre radiografia e TC, concordância Kappa 0,65. As clavículas fraturadas apresentaram medidas semelhantes entre os três métodos (p=0,059) e os testes-T comprovaram que a semelhança foi provocada casualmente ou possíveis erros de medição.

**Conclusão:**

A medição por fita métrica apresentou tendência em superestimação do encurtamento ósseo. A TC apresentou resultados mais confiáveis para o diagnóstico, contudo, a radiografia foi suficiente para tomada de decisão dos cirurgiões e por isso, não é possível descartar a importância deste recurso para FDEMC. ***Nível de Evidência IV; Estudo Caso Controle.***

## INTRODUCTION

Fractures of the clavicle represent between 5 and 10% of all fractures,^1^ it is predominant for the young population whose trauma mechanism is medium to high energy and due to sports and motor vehicle accidents.^2^ The involvement of the midiaphyseal third is present in 70% to 80% of cases, ^3^ and is often associated with bone displacement.^4^

The traditional literature shows a good evolution in non-surgical treatment in fractures of the middle third of the clavicle. ^5^ While surgical treatment was recommended for cases with bone exposure, associated neurovascular injury, floating shoulder, scapulothoracic dissociation, polytraumatized,^6^and presence of bone shortening equal to or greater than 15 to 20 millimeters,^7,8^ the latter being the main predisposing factor for non-bone union, identified in 15% to 21% of cases.^9^

However, current studies have shown failures in non-operative treatment for this type of fracture, especially in those with shortening greater than 20 millimeters.^10^ Therefore, it is essential to standardize the evaluation of clavicle fractures in the therapeutic decision. Bone shortening of the clavicle can be measured through physical examination and imaging tests such as radiography and computed tomography (CT),^11^ the latter resource is considered the "gold standard".^12^ However, CT generates additional costs to care,^13^ and greater exposure of the patient to radiation.

The objective of this study is to analyze the bone shortening in displaced midshaft clavicle fractures (DCMF) and identify the reliability of three different evaluation methods, recommended by physical examination with the aid of a metric tape, digital radiography with anteroposterior incidence and caudocranial axial projection at 20° and CT with 3D reconstructions.

## METHOD

A cross-sectional analytical study was carried out between 2019 and 2020, which evaluated 26 patients seen in a highly complex hospital unit in Orthopedics and Traumatology, who presented unilateral fracture of the middle third of the clavicle with deviation, identified as type II by Robson's classification.^14^ Individuals with bilateral fractures; fractures of the proximal or distal thirds; history of contralateral clavicular fracture were excluded.

Participants were included in the study by signing the Informed Consent Form (ICF) and the study was duly approved by the Research Ethics Committee, under CAAE number: 10751919.8.0000.5412.

### Evaluations

All examinations were bilateral and performed by three experienced examiners. Participants were positioned in orthostatic for evaluation by metric tape.

The imaging tests used in the study were radiography and computed tomography with reconstruction in three dimensions 3D. Imaging tests were carried out with the volunteers in dorsal decubitus, shoulders resting on the table, arms relaxed and parallel to the trunk, and hands positioned on the abdomen. The digital images were evaluated with the aid of the "ruler" tool of the Web Viewer software. For the three evaluation methods, the anatomical measurement points were standardized, considering: Center of the most proximal projection of the sternal end and the center of the most distal projection of the acromial end, forming a rectilinear line.

For the measurements of bone shortening, we considered the differences in length between the clavicles obtained by the three expert examiners, who were blinded and did not have access to each other's data.

### Physical examination with a metric tape (MT)

**Figure 1:**
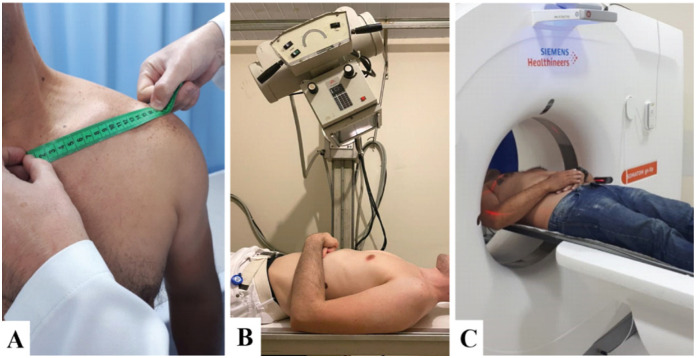
Methods of measuring clavicular length. (A) Physical examination using the metric tape; (B) Radiographic examination with anteroposterior incidence with a caudocranial axial projection of 20°; (C) Examination by computed tomography.

A metric tape (MT) with a millimeter scale was used for bilateral evaluation of clavicle length, for further analysis of differences. The examination was performed with palpation of the sternoclavicular and acromioclavicular joints to identify the acromial and sternal extremities of the clavicles. Then the metric tape, staggered in millimeters (mm), was positioned using the predefined anatomical points and in a rectilinear manner. The tape was malleable to allow adaptation to the contour of the bone deviation (Figure 1A). Surgeons were asked to disregard the joint spaces, requiring more vigorous palpation. Dermographic markings were also not used since this procedure could influence the inter-examiner analyses.

### Digital radiographic examination (X-Ray)

It was performed with anteroposterior incidence with a caudocranial axial projection of 20°, with the patient positioned in horizontal dorsal decubitus and an X-Ray beam oriented to an intermediate point of the clavicles. The distance between the ampoule of the equipment and the patient has been standardized to 1 meter away (Figure 1B).

### Computed Tomography (CT)

Performed in a Siemens device model Somatom Spirit, whose clavicular length was measured by a line between the standardized anatomical points, with the aid of Web Viewer software in 3D axial reconstruction (Figure 1C).

### Statistical Analyses

Initially, the principal components of the non-fractured clavicles (Control) were analyzed to identify the data distribution pattern. Then Friedman's test was applied to analyze the differences in length, with significance index p<0.05 and Kappa coefficient (k) with a confidence interval of 0.95%, to determine the agreement between the evaluation methods from the clavicles without anatomical changes, being considered: k ≤ 0.2 = poor; 0.2 < k ≤ 0.4 = reasonable; 0.4 < k ≤0.6 = good; 0.6 < k ≤ 0.8 = very good; 0.8 < k ≤ 1 = excellent.

After that, the tests were replicated to the bone shortening data present in the fractured clavicles. Finally, data validation occurred through T-tests (p<0.05) for each of the participants, to determine the reliability of the methods. The statistical analyses had been carried through with aid of software "R" version 3.2.2.

## RESULTS

**Figure 2:**
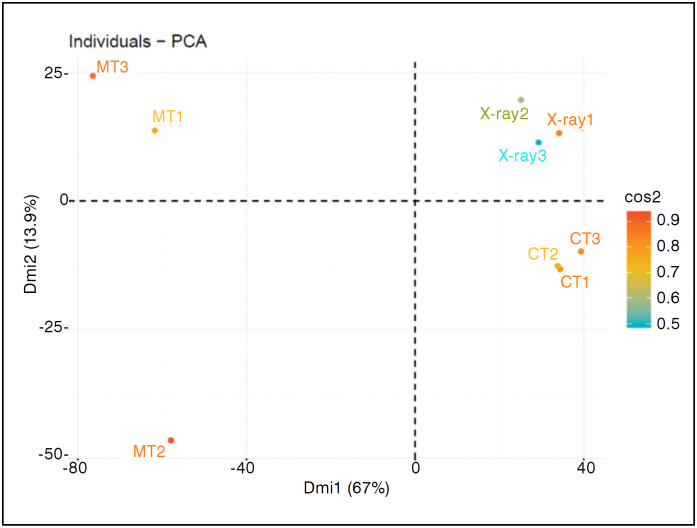
Analysis of main components of the control clavicles in two dimensions (dim) for data distributions of the three examiners. Color patterns indicate the variability of inter-examiner results.

The descriptive analysis of the main components of the non-fractured clavicles (Control) allowed the identification of the data distribution pattern between the three evaluation methods. The radiographic measurements had presented changeable standards between the three examiners, but if they had approached the measures gotten for the computed tomography, whose examination of the image presented greater uniformity of the distribution of the data. The results of the metric tape did not present normal distribution, even after the logarithmic transformation of data by the Box-Cox method (Figure 2).

The non-parametric comparative analysis of the control clavicles by Friedman's test identified a significant difference for MT (p=0.000008), and statistical similarity between X-Ray and CT. The Kappa test demonstrated agreement enters the data of the image examinations (Table 1).

**Table 1:** Statistical comparison between the methods of evaluating the length of the control clavicles. (ns) Not significant.

Comparison between methods	Statistical test	
	Friedman	Kappa
MT versus X-Ray	<0.05	0.45
MT versus CT	<0.05	0.34
X-Ray versus CT	ns	0.65

**Figure 3:**
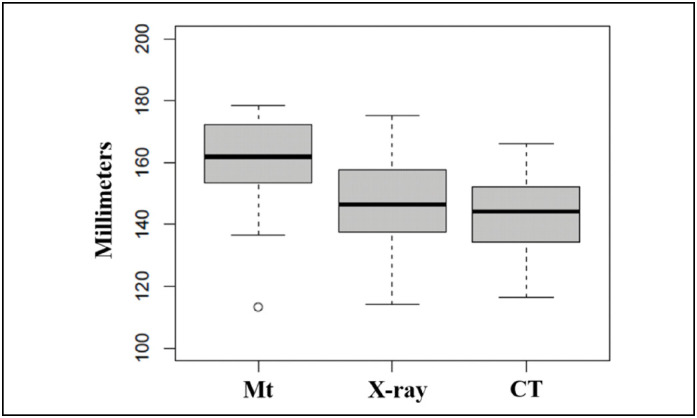
Distribution of mean clavicle lengths controlled for the three assessment methods.

Measurement by metric tape showed a tendency to overestimate bone length (Figure 3)

After identifying the pattern of distribution, agreement, and differences between the evaluation methods in clavicles without biological changes (Control), the study directed the analysis to the differences in bone length identified in the fractured clavicles compared to the control side.

**Figure 4:**
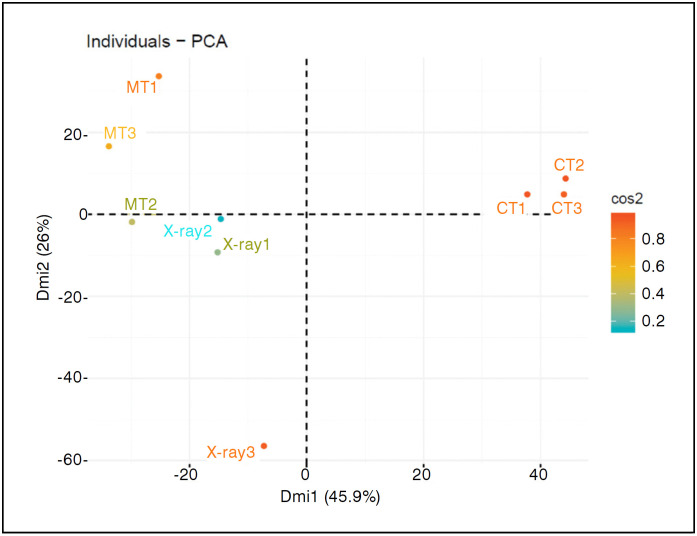
Analysis of main components of bone shortening of fractured clavicles, in two dimensions (dim) for data distributions of the three examiners. Color patterns indicate the variability of inter-examiner results.

The descriptive analysis of the main components of the differences in bone length showed a different distribution pattern, approximating the radiographic measurements to the measurements of the metric tape (Figure 4).

When comparing differences in bone length between the three methods of measurement, the test of Friedman did not identify significant differences (p=0,059). The average clavicular length and bone shortening are shown below (Table 2).

**Table 2:** Averages of clavicular length and absolute (millimeters) and relative (percentage) bone shortening.

Exam	Non-fractured clavicle (mm)	Fractured clavicle (mm)	Bone shortening (mm)	Relative bone shortening (%)
MT	165.5±16.3	154.7±15.2	10.8±6.4	6.4
X-Ray	151.5±16.5	144.5±16.0	7.1±7.2	4.5
CT	145.2±13.2	139.1±14.2	6.1±9.9	4.1

**Figure 5:**
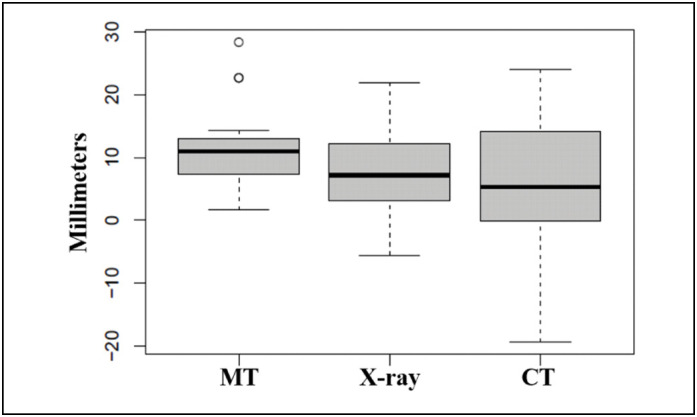
Distribution of mean differences in clavicular length for the three assessment methods

Although bone shortening was similar among the clinical evaluation methods, great variability of the results was found for the computed tomography examination (Figure 5).

The variability was caused by a higher incidence of elongation of the clavicle present in both imaging exams and especially in CT. This result generated the hypothesis that the use of the control side to measure the difference in clavicular length might not be a good alternative.

This preliminary result required an individual statistical evaluation for each of the 26 study participants with the application of T-tests to determine whether the differences between the clavicles were caused by biological factors, by chance, or measurement error. From this point, the relative frequencies of the presence of bone shortening before and after statistical validation were analyzed (Table 3).

**Table 3:** Frequency of bone shortening after statistical validation.

Exam	Before validation of test	After the validation test	Variation Percentage
MT	26	5	80.8%
X-Ray	20	13	65.0%
CT	19	17	10.5%

The only method that kept valid from the initial analysis was the CT results. Both the measurements with measuring tape and radiographs showed significant changes, indicating that the difference was caused by the chance or lower accuracy of the method.

The evaluation of bone shortening by measuring tape, in addition to underestimating the measurements, made the examiners present a tendency to always seek bone shortening.

## DISCUSSION

In this study, different measurement methods for bone shortening in FMD were compared, using physical examination with the aid of a tape measure and two other methods composed of imaging exams, with anteroposterior axial radiographs at 20° with caudocranial projection and CT with 3D reconstructions.

As described by Smekal *et al*,^13^ low reliability was identified for evaluation with metric tape for bone length measurement, which can be influenced by soft tissue coverage, while radiographs and tomography showed comparable repeatability. In total, the 26 individuals evaluated in this study showed more bone shortening when evaluated by measuring tape than by imaging exams.

A variety of techniques for the evaluation of the DMCF exists, but it does not have a consensus on an optimum method or standardization for the accomplishment of the image examinations.^14,15^ Two concepts are more accepted to evaluate the shortening: measurement of the difference in bone length between the clavicles or overlapping the fragments.^15^ Although the first concept is described as more reliable,^4^ it is also subject to anatomical differences between the clavicles, present between 28.5% and 30% of the population and which may be greater than 5 mm in length,^16^ in addition to the influence of radiographic incidence,^13^ and patient positioning.^17,18^ In this study, we recommend the method of evaluating the differences between the fractured clavicle and the contralateral one, as it is the only method that could be reproduced in evaluations by metric tape. The study of Archer *et al*,^11^ had been evaluated 22 patients with DMCF and although the excellent correlation between the examiners, did not have agreement enters the measures gotten for conventional x-rays AP and TC, in virtue of the error of measurement of 6,96 centimeters identified in the x-rays. It is also described that radiographic films can favor the overestimation of bone shortening on average 8.2 mm concerning CT.^12^ Corroborating these results, the present study also noted the tendency of examiners to quantify greater bone shortening, however, an average difference of only 1 mm was found between digital radiographic images and CT with 3D axial reconstructions, evidencing the importance of standardization of radiographic examination in clinical practice.

When evaluating initially the methods of measurement from the measures of clavicle control, we do not evidence significant differences between the image examinations. The statistical similarity was also present for bone shortening measurements of fractured clavicles. However, the confirmation of the results with the application of T-tests for each individual showed that the frequency of shortenings on tomographic examination remained similar before and after statistical validation, while the same did not occur for the X-Ray. Despite the very good agreement between the imaging tests, the radiographic evaluation was more subject to differences because it was less accurate.

It is important to highlight that of the twenty six individuals evaluated, sixteen should receive conservative treatment according to the evaluations by metric tape and radiographs, and in only one case CT was able to change the opinions of surgeons to a surgical approach, due to the complexity and comminution of the fragments. For this reason, we cannot say whether the differences in shortening observed between both imaging exams are relevant in clinical practice by experienced surgeons.

Some limitations to this study must be taken into consideration: a limited number of literature on the use of the metric tape to quantify bone shortening in DMCFs; the absence of different radiographic projections for comparison purposes; impossibility of intra-examiner evaluation, since it is an emergency care service for orthopedic trauma, which made it impossible to collect measurements at different times; antalgic position of patients and the difficulty of palpation of bone structures in individuals with overweight or presence of swelling and abrasions in the anatomical areas used as reference points; comparison of the physical examination performed in the orthostatic position against the image evaluations that were performed with the patient in dorsal decubitus, generating variability in the results.

## CONCLUSION

The evaluation of bone shortening with the aid of MT showed less reliability, greater variability and a tendency to overestimate measurements. Although measurements maked by radiography also showed variability, concordance was verified with the data obtained by 3D computed tomography, whose differences were not influence the orthopedic surgeons’ treatment decision. For this reason, the importance of radiographic evaluation for the evaluation of DMCF cannot be ruled out. Statistical validation proved that the measurement of bone shortening by means of CT with 3D image reconstructions is less subject to measurement errors and overestimation of bone shortening, being the most reliable resource in this study.
